# Use of an endoscopic virtual ruler based on the fiber laser principle and artificial intelligence technology

**DOI:** 10.1055/a-2409-0070

**Published:** 2024-09-19

**Authors:** Yaxian Kuai, Shiwei Zhou, Bin Sun, Xu Wang, Youwei Xiao, Aijiu Wu, Derun Kong

**Affiliations:** 1 36639 Department of Gastroenterology, The First Affiliated Hospital of Anhui Medical University, Hefei, China; 2 601409 School of Software, University of Science and Technology of China School of Software Engineering, Hefei, China; 3 Research and Development Department, Hefei Zhongna Medical Instrument Co. Ltd, Hefei, China

The accurate measurement of lesion size during endoscopic procedures is of paramount importance. It not only informs the assessment of disease risk, but also guides the selection of appropriate surgical interventions and provides a foundation for effective treatment monitoring 1 . Current endoscopic measurement techniques, such as visual inspection, the tension clamp method, and the implantable instrument ruler, still exhibit certain limitations 2 . To address this challenge, researchers have explored the development of an endoscopic virtual ruler based on fiber laser principles and artificial intelligence (AI) technology, which aims to enable simple and precise real-time measurement of various types of lesions.

In this innovative model, a laser-based approach is employed to precisely measure the size and distance of targets 3 . Leveraging a medical endoscope, fiber-coupled laser, laser collimator, and an advanced AI algorithm, the system generates laser spots that appear at varying size at different distances ( Fig. 1 ). By analyzing the spot area in the captured image and correlating it with the known scale bar, the system is able to accurately estimate the distance to the object and calculate its actual size. This end-to-end solution streamlines the measurement process during medical procedures, enabling healthcare professionals to make informed decisions based on reliable data-driven insights. The integration of cutting-edge laser technology and intelligent software algorithms underscores the continuous advancements in medical imaging and diagnostics.

**Fig. 1:**
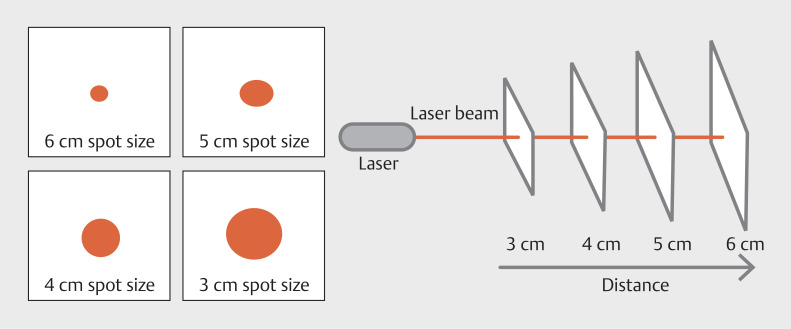
Schematic diagram of the endoscopic virtual ruler construction.

Video 1 shows the novel model being used to accurately size a large raised polyp, a laterally spreading polyp, and an early gastric cancer ( Fig. 2 ).

**Fig. 2:**
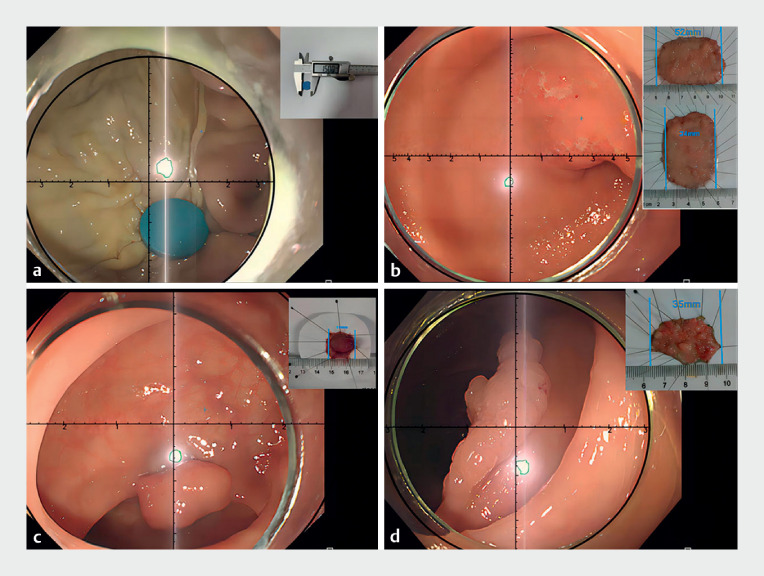
Examples of measurements made using the virtual ruler for: **a** a round foreign body in an ex vivo pig stomach; **b** an early antral cancer; **c** a sigmoid colon polyp; **d** a laterally spreading polyp in the ascending colon.

An introduction to the endoscopic virtual ruler, including its demonstration on an ex vivo pig stomach and three different lesions in patients.Video 1

Endoscopy_UCTN_Code_TTT_1AQ_2AI

## Notes

Endoscopy E-Videos https://eref.thieme.de/e-videos
*E-Videos* is an open access online section of the journal *Endoscopy* , reporting on interesting cases and new techniques in gastroenterological endoscopy. All papers include a high-quality video and are published with a Creative Commons CC-BY license. Endoscopy E-Videos qualify for HINARI discounts and waivers and eligibility is automatically checked during the submission process. We grant 100% waivers to articles whose corresponding authors are based in Group A countries and 50% waivers to those who are based in Group B countries as classified by Research4Life (see: https://www.research4life.org/access/eligibility/ ). This section has its own submission website at https://mc.manuscriptcentral.com/e-videos .
